# From Infection to Anxiety: A Sequential Model Linking Latent Toxoplasmosis to Psychological Distress via Health and Stress

**DOI:** 10.1111/sjop.70085

**Published:** 2026-02-26

**Authors:** Jaroslav Flegr, Ashkan Latifi, Šárka Kaňková

**Affiliations:** ^1^ Laboratory of Evolutionary Biology, Department of Philosophy and History of Sciences, Faculty of Science Charles University Prague Czech Republic

**Keywords:** *Borrelia*, latent toxoplasmosis, manipulation hypothesis, physical health

## Abstract

Identifying the drivers of chronic stress is crucial for understanding its impact on mental health. Latent toxoplasmosis, a widespread parasitic infection, has been linked to various psychological changes. The Stress‐Coping Hypothesis proposes that at least some of these changes are consequences of chronic stress arising from the infection's negative impact on physical health, rather than direct parasitic manipulation. To test this mediational pathway, we surveyed 1768 individuals previously tested for toxoplasmosis or borreliosis, using the Perceived Stress Scale and the State–Trait Anxiety Inventory. As predicted, *Toxoplasma*‐infected individuals reported significantly poorer physical health and higher levels of perceived stress and anxiety. Crucially, path analysis revealed a clear sequential mechanism: toxoplasmosis was directly associated only with poorer physical health, which in turn predicted higher perceived stress, which subsequently predicted increased anxiety. This specific, stress‐mediated pathway was absent in the control group of individuals with borreliosis, providing strong evidence that the psychological effects of this common infection are side effects of illness‐induced chronic stress. These findings offer a mechanistic model of how a chronic physical health burden translates into significant psychological distress and highlight the importance of considering latent infections as a contributor to the global burden of stress‐related disorders.

## Summary

- Latent *Toxoplasma gondii* infection is associated with increased levels of perceived stress and anxiety in the general population.
- Path analysis revealed a sequential mechanism: toxoplasmosis impairs physical health, which increases perceived stress, leading to elevated anxiety.
- This stress‐mediated pathway was specific to toxoplasmosis and not observed in individuals with borreliosis.
- The findings support the Stress‐Coping Hypothesis, suggesting that some behavioral effects of toxoplasmosis are side effects of chronic health‐related stress, not direct manipulation.
- Given the high global prevalence of toxoplasmosis, these results highlight its underestimated contribution to the burden of stress‐related disorders.

## 1 Introduction

Toxoplasmosis is a zoonotic parasitic infection caused by the obligate intracellular protozoan *Toxoplasma gondii*. It is estimated that currently about one‐third of the population in both developed and developing countries is infected, with the infection, characterized in its later stage by the presence of dormant stages in the bodies of infected individuals, likely being lifelong (Pappas et al. 2009; Tenter et al. 2000). *Toxoplasma gondii* undergoes sexual reproduction in the intestinal epithelial cells of its definitive host, a feline. The resulting oocysts are shed in the host's feces and become infectious after several days of sporulation in the environment. Intermediate hosts—typically rodents but also other warm‐blooded animals—become infected after ingesting these oocysts. In the bodies of intermediate hosts, sporozoites released from ingested oocysts invade cells and transform into tachyzoites, which then begin to rapidly asexually reproduce in various tissues and organs, including the brain, heart, and skeletal muscles. Over time, this process leads to the formation of tissue cysts, the transformed host cells, containing bradyzoites, marking the onset of lifelong latent infection.

*Toxoplasma* spreads both vertically—from infected mothers to fetuses—and horizontally, through ingestion of oocysts or infected prey. Congenital infection often causes severe developmental defects or miscarriage (Pappas et al. 2009; Tenter et al. 2000). Postnatally acquired toxoplasmosis was long considered asymptomatic in immunocompetent hosts. However, studies over the past 30 years using both animal and human models have revealed multiple effects on infected individuals, including behavioral changes that may represent adaptive strategies to enhance transmission (Abdulai‐Saiku et al. 2017; Saadatnia and Golkar 2012; Webster et al. 1994; Wohlfert et al. 2017). These manipulations can alter the host's risk‐taking behavior, social interactions, or even cognitive processes, potentially increasing the likelihood of predation and thus facilitating the parasite's life cycle (Abdulai‐Saiku et al. 2017; Pardo Gil et al. 2023; Saadatnia and Golkar 2012; Webster et al. 1994; Wohlfert et al. 2017). Notably, one such manipulation involves the intermediate host developing an attraction to the urine odor of the definitive host, effectively drawing it closer to its natural predator. This phenomenon has been observed in *Toxoplasma*‐infected rodents (Berdoy et al. 2000), chimpanzees (Poirotte et al. 2016), and humans (Flegr et al. 2011). In modern humans, who are no longer primary prey for felines, the effects of infection with this parasite have also been observed in various other domains, such as personality (Flegr et al. 2023, 1996), psychopathology (Flegr and Horáček 2020; Rahiminezhad et al. 2024), cognition (de Haan et al. 2021), and behavior (Lerner et al. 2020; Lindová et al. 2006).

Research on latent toxoplasmosis and personality suggests that infection affects men and women in opposite ways. Infected women tend to show higher cooperativeness, trust, superego strength, and extraversion, whereas infected men typically score lower on these traits (Flegr and Hrdý 1994; Flegr et al. 1996; Lindová et al. 2006). As a potential explanation for this observation, researchers have proposed a psychological rationale focusing on the distinct coping strategies employed by men and women in response to prolonged stress caused by infection (Lindová et al. 2010, 2006). Men generally rely on rational and individual coping strategies, whereas women more often use emotional or avoidance‐based strategies and seek social support during stress (Kelly et al. 2008; Matud 2004). In the study by Lindová et al. (2006), the health decline caused by prolonged stress from infection is suggested to lead *Toxoplasma*‐infected individuals to adopt gender‐specific coping strategies. These strategies might explain the observed divergent personality traits between infected males and females.

The lifelong presence of bradyzoite cysts, primarily in neural tissue, necessitates a persistent host immune response, which can serve as a chronic stressor. *Toxoplasma* infection triggers a sustained, low‐grade inflammatory state, characterized by a Th1 immune response and the production of pro‐inflammatory cytokines such as Interferon‐gamma and Tumor Necrosis Factor‐alpha (Latifi and Flegr 2025). This chronic inflammation directly impacts the Central Nervous System, as these cytokines can cross the blood–brain barrier. For example, research on neurodegenerative diseases shows that IFN‐γ can activate the kynurenine pathway, diverting tryptophan, a precursor to serotonin, towards the production of neurotoxic metabolites like quinolinic acid (Lovelace et al. 2016). This disruption to neurotransmitter systems is well‐known to be associated with mood and behavioral changes. Chronic cytokine signaling acts as a persistent stress signal, stimulating the Hypothalamic–Pituitary–Adrenal (HPA) axis, the body's central stress response system. This results in the prolonged secretion of glucocorticoids such as cortisol (O'Connor et al. 2000; Silverman et al. 2005). Sustained cortisol exposure can lead to neuroplastic changes in brain regions critical for emotion regulation, such as the hippocampus and the amygdala (McEwen et al. 2016). These neuroimmunological changes, chronic inflammation, neurotransmitter dysregulation, and HPA axis hyperactivity likely constitute the biological basis for the prolonged stress in *Toxoplasma* infected individuals. The divergent personality changes observed between infected men and women may reflect interactions between these *Toxoplasma*‐related alterations and established sex differences in HPA‐axis reactivity and immune responses (Rao and Androulakis 2017), potentially contributing to differences in psychological coping.

Latent toxoplasmosis is indeed found to negatively affect the physical health of the infected individuals (Flegr and Escudero 2016; Flegr et al. 2014). What remains unclear is how this infection‐related impairment translates into psychological outcomes. The Stress–Coping Hypothesis proposes that chronic infection acts as a long‐term stressor, eliciting different coping strategies in men and women, which may secondarily manifest as sex‐specific behavioral and personality changes. Yet direct empirical tests of this hypothesis, especially studies assessing perceived stress with validated psychometric instruments, are, to our knowledge, currently lacking. Addressing this gap is essential to test whether latent toxoplasmosis influences stress primarily indirectly—via impaired physical health rather than through a direct effect on psychological states.

A typical behavioral symptom of chronic stress is anxiety (Coppola and Spector 2009; Endler and Parker 1990; Hussenoeder et al. 2022; Qin et al. 2015; Smith et al. 2015). Therefore, we predicted that *Toxoplasma*‐infected individuals would show higher anxiety levels than uninfected controls. In line with that, laboratory mice in the latent stage of *Toxoplasma* infection exhibit anxiety‐like behavior (Bay‐Richter et al. 2019), and an increased incidence of Generalized Anxiety Disorder has been reported in *Toxoplasma*‐infected humans (Akaltun et al. 2018; Flegr and Horáček 2020; Suvisaari et al. 2017). Surprisingly, data demonstrating an association between latent toxoplasmosis and increased anxiety as a trait in the nonclinical population are scarce. The sole study indicating increased anxiety (along with depression and obsession) in *Toxoplasma*‐infected individuals, conducted on a population of about 3600 subjects, did not measure personality traits anxiety with psychological questionnaires but relied on participants' subjective ratings of the intensity of suffering from specific neuropsychiatric symptoms (depression, mania, phobia, anxiety, and obsessions) on a 100‐point scale (Flegr and Horáček 2020).

Another zoonotic disease, although much less comprehensively studied than toxoplasmosis in relation to potential infection‐induced behavioral changes, is borreliosis. Borreliosis is caused by the bacterium *Borrelia burgdorferi* (Nadelman and Wormser 1998). *Borrelia* is transmitted through tick bites and can persist for years in an infected human's brain, heart, liver, and kidney, even after extensive antibiotic therapy (Sapi et al. 2019), suggesting its potential for a life‐long latency. The seroprevalence of borreliosis is reported to be 13.6% in Western Europe, 11.1% in Eastern Europe, 4.2% in Northern Europe, and 3.9% in Southern Europe by a meta‐analysis of the related studies which used two‐tier testing from 2005 to 2020 (Burn et al. 2023). While latent *Borrelia* infection, unlike the much rarer neuroborreliosis, was historically considered to be almost asymptomatic, it is essential to acknowledge recent findings from two studies indicating that borreliosis can lead to deteriorated health in infected individuals (Flegr and Horáček 2018; Flegr et al. 2023). Given the nature of its life cycle, *Borrelia* is not expected to have evolved mechanisms that specifically alter host behavior or psychological state to facilitate its transmission. Moreover, it was demonstrated that, unlike latent toxoplasmosis, latent *Borrelia* infection manifests only in poorer physical health, not in mental health (Flegr and Horáček 2018; Flegr et al. 2023).

The main aim of this study was to test the Stress‐Coping Hypothesis by examining perceived stress levels in *Toxoplasma*‐infected individuals. A secondary aim was to use path analysis to separate direct effects of infection on stress and anxiety from those mediated by physical health. Data were collected from 1768 nonclinical internet users previously tested for toxoplasmosis using an online survey that included the Perceived Stress Scale and the State–Trait Anxiety Inventory. To control for possible reporting bias, the presence of *Borrelia* infection was used as a negative control, since both infections were self‐reported but only one (toxoplasmosis) was expected to affect stress levels.

## 2 Material and Methods

### 2.1 Participants

Participants were recruited using a Facebook‐based snowball sampling approach (Flegr et al. 2022). Invitations to an online survey about well‐being and discomfort during the COVID‐19 pandemic were posted on several Facebook pages/groups, and respondents were encouraged to share the link further (friend‐of‐friend diffusion). This method has known limitations for representativeness: it reaches only individuals with internet access—disproportionately those who use social media—and can introduce demographic skew (e.g., towards younger, more educated, and urban users) as well as self‐selection of participants with greater interest in health or psychology. At the same time, chain referral beyond immediate friends helps the invitation propagate across multiple social circles, yielding a broad and heterogeneous sample. To mitigate bias, we controlled for key covariates in all multivariable analyses; nevertheless, the sample should not be considered a random cross‐section of the population.

The introductory page of the approximately 10‐min survey informed participants about its purpose and the topics covered—demographic information, personality, life satisfaction, and current opinions. They were also assured of anonymity, the exclusive research purpose of the collected data, its confidentiality, the voluntary nature of participation, and the option to withdraw at any time by closing the webpage. Informed consent was obtained by participants clicking a button on the page. Participants were not financially compensated for their participation in the study. Instead, they received their personal results from the STAI‐X2 and PSS questionnaires, along with histograms showing the distribution of these results among participants from the previous similar study. The survey, part of the project “Influence of Biological, Socioeconomic, and Psychological Factors on People's Behavior and Attitudes in Relation to Epidemiology” was approved by the Institutional Review Board of the Faculty of Science, Charles University and conducted following all relevant guidelines and regulations. The final version was launched from August 28, 2020, through the end of the year, with most of the 7214 respondents completing it by the end of October 2020.

### 2.2 Questionnaires

Two standardized questionnaires were employed for collecting data on anxiety and stress in our sample. State‐Trait Anxiety Inventory X‐2 (STAI‐X2), re‐standardized by (Heretik et al. 2009) in an adult Slovak population, was implemented to evaluate the anxiety trait in the participants. Heretik et al. reported alpha reliability coefficients of 0.85 through 0.88 for this questionnaire. STAI‐X2 is composed of 20 items scaled on a 4‐point scale (almost never, sometimes, often, almost always). Higher scores on STAI‐X2 indicate higher levels of anxiety trait. On the STAI‐X2, the lowest possible score is 20, and the highest possible score is 80. Stress was measured via implementation of the Perceived Stress Scale (PSS), which nests 10 items scaled on a 5‐point scale (never, almost never, sometimes, quite often, very often). On the PSS, 0 is the lowest possible score, and 40 is the highest possible score, with the higher scores denoting higher levels of perceived stress. The PSS was originally developed by Cohen et al. (Cohen et al. 1983), who reported alpha reliability coefficients of 0.84, 0.85, and 0.86 in the three samples that they used in their studies. We also assessed the reliability of these questionnaires in our sample for all participants, and also for men and women separately. The reliability coefficients of McDonald's omega total and Cronbach's alpha for STAI‐X2 for all participants were 0.94 and 0.93, respectively. They proved 0.94 and 0.93 for women, and 0.93 and 0.92 for men on this questionnaire, respectively. We also calculated the McDonald's omega total and Cronbach's alpha for PSS, which resulted in the coefficients of 0.90 and 0.88 for all participants, 0.90 and 0.88 for women, and 0.89 and 0.87 for men, respectively. Overall, these results demonstrated acceptable reliabilities for these instruments in our study. In addition, STAI‐X2 exhibited higher reliability coefficients for all participants, men, and women when compared with PSS. On the whole, STAI‐X2 and PSS had slightly higher reliability in the women sample compared to the men sample.

Both the Perceived Stress Scale (PSS) and the State–Trait Anxiety Inventory (STAI‐X2) were administered in Czech, which is mutually intelligible with Slovak, allowing participation of both Czech and Slovak respondents. These instruments were selected because they are well‐validated, widely used in behavioral and epidemiological research, and suitable for online administration. Regarding measurement validity, previous research has established the construct validity of both instruments within the Czech and Slovak populations. The STAI‐X2 was extensively validated during its restandardization on a Slovak adult sample, which demonstrated strong convergent validity with other measures of emotionality and temperament (Heretik et al. 2009). Similarly, the Czech version of the PSS has been utilized and validated in various clinical and non‐clinical contexts, showing a stable two‐factor structure and significant correlations with related psychological constructs such as anxiety and depression (Plháková 2005). As no official Czech standardization exists, raw (non‐standardized) questionnaire scores adjusted for sex and age were used in all analyses rather than normative values.

In the final part of the questionnaire, participants were asked to indicate whether they had toxoplasmosis or borreliosis, with explicit instructions to base their responses solely on laboratory test results. They were reminded that toxoplasmosis is a parasite of cats that is especially dangerous for pregnant women, and also that borreliosis is a tick‐borne bacterial disease transmitted through the bite of ticks. Accordingly, in this regard, they could choose one of the three options available to them (1) positive—I was tested and the result of the test was positive, (2) negative—I was tested and the result of the test was negative, or (3) I do not know, I am not sure. No new serological testing was performed in this study. Instead, infection status was based on self‐reported prior laboratory diagnoses. This approach can better indicate latent infection over long time spans, because antibody titers decline with time since infection and may fall below diagnostic thresholds. This scenario is more common in older participants, who are likelier to have been infected many years earlier (see Discussion).

Our participants also self‐rated their physical health using a scale of 0 to 100, where 0 meant “very bad” and 100 meant “very good” for their physical health today. Participants also disclosed their sex assigned at birth, their age, and the size of their place of residence, which was rated on a 7‐point ordinal scale. The overarching aim of the entire project was to explore the effects of the pandemic and lockdowns on the well‐being of the general Czech population; therefore, the questionnaire included numerous questions unrelated to the current study.

### 2.3 Statistical Analysis

#### 2.3.1 Inclusion and Exclusion Criteria

All respondents who completed the full questionnaire and provided information about their laboratory test results for toxoplasmosis or borreliosis (positive or negative) were included in the study. Participants were required to be at least 14 years old; in practice, the youngest participant who completed the survey was 17 years old. Individuals who discontinued the questionnaire before reaching the infection‐related questions were excluded. For each statistical analysis, all individuals who answered the relevant items were included.

We conducted the Shapiro–Wilk test and examined data distribution plots to assess the normality assumption. As mild deviations from normality were detected for stress and anxiety scores, and because the numbers of *Toxoplasma*‐infected and uninfected participants differed substantially, we complemented parametric analyses with non‐parametric methods. Parametric procedures such as ANCOVA and MANCOVA are generally robust to moderate violations of normality and unequal group sizes in large samples; nonetheless, non‐parametric tests (Wilcoxon rank‐sum tests and partial Kendall's Tau correlations) were used as assumption‐light robustness checks. Importantly, parametric and non‐parametric approaches yielded nearly identical results throughout, indicating that the findings were not driven by distributional shape or group‐size imbalance. Categorical data, namely sex and infection status, were cross‐tabulated, and Chi‐squared tests were used to uncover their potential associations.

We conducted both multivariate and univariate analyses of covariance (MANCOVA and ANCOVA) using Type III sum of squares to investigate the effects of infection and sex, as well as their interactions, on anxiety and stress, with age as a covariate. This approach allowed us to accurately assess each factor's unique contribution by adjusting for the variance explained by other variables in the model, ensuring robust analysis in potentially unbalanced designs or when multiple covariates are involved. We also considered the size of the place of living as a potential confounder. However, AIC comparisons showing minimal difference between simpler and complex models led us to exclude the living place size from our final analysis. The effects of sex and infection on age, physical health, anxiety, and stress were further analyzed using univariate non‐parametric Wilcoxon rank‐sum tests and multivariate non‐parametric partial Kendall's Tau correlation tests. Covariates age, sex, and physical health were controlled in the partial Kendall's Tau correlation tests. Sex and age were the only mandatory questionnaire items; participants could not proceed without answering them. Accordingly, these covariates were available for all respondents and contained no missing data. Regarding multiplicity, we did not apply a global correction because the analysis targeted two pre‐specified outcomes (perceived stress and trait anxiety) with *Toxoplasma* infection as the focal predictor and *Borrelia* as a negative control. However, the associations of *Toxoplasma* with perceived stress and anxiety remained significant even under a Bonferroni adjustment for two outcomes.

Path analysis was employed for an in‐depth exploration of the relationships among various variables. This method allowed for the differentiation between direct and indirect effects of the primary interest variables, specifically toxoplasmosis and borreliosis. It further facilitated the description and distinction of the mediating and confounding effects of physical health and age. To evaluate collinearity among predictors, we examined variance inflation factors and pairwise associations; no evidence of problematic multicollinearity was detected. In path models, inspection of the standardized covariance matrix and residual correlations indicated stable estimation without near‐singularities. For all variance analysis models, the VIF values were close to 1, and correlations among predictors were below 0.3, indicating no multicollinearity concerns. In the path models (both overall and by sex), the majority of correlations among observed variables were below 0.1, with none exceeding 0.5 except for those between stress and anxiety, which ranged from 0.70 to 0.87 across models. Statistically, such correlation magnitudes are acceptable, and conceptually, stronger associations between stress and anxiety are expected. Finally, the residual correlations were zero in all models, indicating stable estimation, absence of near‐singularities, and just‐identified models allowing for the interpretation of path parameter estimates, which are directly related to the main objective of our study: the estimation of prespecified direct and indirect paths. Confidence intervals for the path coefficients were computed using the Delta Method. This method approximates how the estimates would vary in repeated samples by applying a first‐order Taylor series expansion to the model parameters. Using the asymptotic variance–covariance matrix (valid under large‐sample assumptions) from maximum likelihood estimation, we constructed 90% Wald‐type confidence intervals for each path.

The number of participants included in each of these statistical tests is shown in Table S1. Furthermore, we used R (version 4.3.0) for doing descriptive statistics, Wilcoxon rank‐sum test, and path analysis (via packages lavaan and semPlot). Non‐parametric Kendall Tau partial correlations were conducted using Explorer 1.0 (Flegr and Flegr 2021). We also used SPSS (version 26) for MANCOVA and ANCOVA modeling.

All data are available at figshare https://doi.org/10.6084/m9.figshare.25188788.v1.

### 2.4 Technical Notes

(1) Except for the Section Discussion, we use the terms “toxoplasmosis” and “borreliosis” as a shorthand for scoring *Toxoplasma*‐ or *Borrelia*‐seropositive in standard laboratory tests for latent toxoplasmosis. (2) Throughout the manuscript, we use the technical term “effect” in a sense used in statistics that is, as referring to the difference between the true population parameter and the null hypothesis value. The Discussion Section is the only place where we discriminate between cause and effect and use “effect” in its nontechnical sense.

## 3 Results

### 3.1 Descriptive Statistics (Sample Characteristics)

**FIGURE 1:**
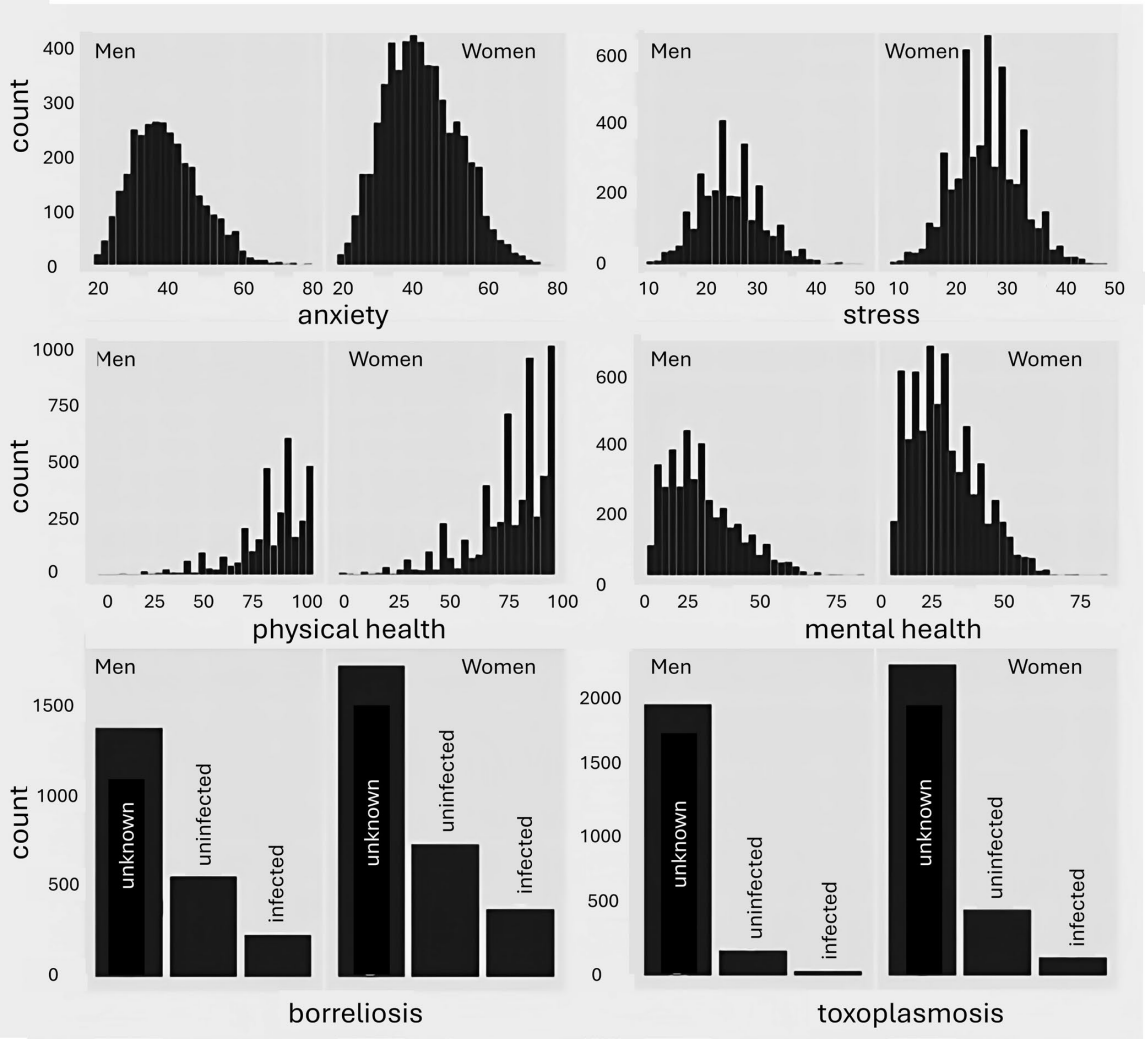
Data distribution of physical health, anxiety, stress, borreliosis, toxoplasmosis, and age for men and women. Upper panels show histograms of anxiety and stress scores separately for men and women. Middle panels display the distributions of self‐rated physical health and age. Lower panels summarize the numbers of participants who were infected, uninfected, or unaware of their infection status for borreliosis (left) and toxoplasmosis (right). The figure illustrates that anxiety and stress levels are higher on average in women than in men, whereas self‐rated physical health is slightly better in men. The age distributions are comparable between sexes, with most participants between 20 and 40 years old. As expected, a large proportion of respondents did not know their infection status, while confirmed infections were less frequent and more common for borreliosis than for toxoplasmosis.

The initial dataset comprised 7214 individuals. For this study, we focused on the 1768 subjects who reported a test result for toxoplasmosis or borreliosis. We divided them into three groups: the Toxoplasmosis Test Group (698 subjects tested for toxoplasmosis: 172 men, 526 women), the Borreliosis Test Group (1584 subjects tested for borreliosis: 650 men, 934 women), and the Dual Test Group (514 subjects tested for both pathogens: 148 men, 366 women). The Dual Test Group was utilized only for the MANCOVA and ANCOVA analyses that addressed both infections in a single model to assess their potential interaction. Descriptive statistics for the overall dataset are illustrated in Figure 1, while the descriptive statistics for the aforementioned groups are detailed in Table 1.

**TABLE 1:** Descriptive statistics for Toxoplasmosis, Borreliosis, and Dual Test groups.

	Dual Test Group									
	Toxoplasmosis					Borreliosis				
	Infected (*N* = 86)		Uninfected (*N* = 428)		*p*	Infected (*N* = 106)		Uninfected (*N* = 408)		*p*
	Mean	SD	Mean	SD		Mean	SD	Mean	SD	
Age	33.83	11.04	32.33	10.71	0.238	36.58	11.47	31.54	10.34	**0.0001**
Physical health	78.13	19.02	83.6	17.05	**0.007**	80.89	16.55	83.15	17.72	**0.037**
Anxiety	43.85	11.33	40.44	10.51	**0.016**	41.06	10.31	41.00	10.83	0.859
Stress	27.05	6.1	25.79	6.5	0.123	26.66	6.32	25.83	6.48	0.235

	Toxoplasmosis Test Group					Borreliosis Test Group				
	Infected (*N* = 146)		Uninfected (*N* = 552)		*p*	Infected (*N* = 527)		Uninfected (*N* = 1057)		*p*
	Mean	SD	Mean	SD		Mean	SD	Mean	SD	
Age	32.6	10.39	31.29	10.61	0.124	36.12	11.83	32.65	11.37	**0.0001**
Physical health	79.3	18.53	83.1	17.45	**0.013**	81.19	16.92	82.36	16.91	0.103
Anxiety	42.88	10.41	40.75	10.62	**0.026**	41.00	9.79	40.62	10.18	0.256
Stress	26.84	6.15	26.10	6.64	0.158	26.11	6.03	25.74	6.17	0.185

*Note: Toxoplasma*‐infected participants showed slightly poorer physical health and higher anxiety than uninfected controls. For *Borrelia*, groups differed mainly in age, with minimal differences in health, stress, or anxiety. The table presents the descriptive statistics (means and standard deviations) and the *p*‐values of the nonparametric Wilcoxon rank‐sum test comparing infected and uninfected participants. Results significant at *p* < 0.05 are highlighted in bold. For detailed results of the tests see Table S2 and for more accurate results, refer to Table 4, which displays the outcomes of multivariate nonparametric tests (partial Kendall correlation test controlled for sex and age, or sex, age, and physical health).

Cross‐tabulation of toxoplasmosis and sex (all men = 172 [24.64%], all women = 526 [75.36%]; infected men = 28 [16.27%], infected women = 118 [22.43%]) showed a non‐significant trend towards a higher prevalence of toxoplasmosis in women (*χ* ^2^_(1)_ = 2.967, *p* = 0.084). The same analysis for borreliosis and sex (all men = 650 [41.04%], all women = 934 [58.96%]; infected men = 206 [31.69%], infected women = 321 [34.36%]) yielded no effect of sex on prevalence of borreliosis (*χ* ^2^_(1)_ = 1.236, *p* = 0.266). In the Dual Test Group that is, in participants who reported data on both infections, the prevalence of *Borrelia* infection was higher among individuals infected with *Toxoplasma* (34 subjects [39.53%]) compared to those not infected with *Toxoplasma* (72 subjects [16.82%]). This disparity was statistically significant (*χ* ^2^_(1)_ = 22.566, *p* < 0.0001).

### 3.2 Stress and Anxiety in Infected and Uninfected Subjects (Group Comparisons and Multivariate Analyses)

#### 3.2.1 Association Between Toxoplasmosis and Borreliosis and Anxiety and Stress (MANCOVA and ANCOVA Results)

The results of Box's test for equality of covariance matrices and Levene's test for equality of error variances for stress were satisfactory for the Toxoplasmosis Test Group, Borreliosis Test Group, and the Dual Test Group. The analyses indicated a breach of the assumption of equal error variances for anxiety within the Borreliosis Test Group, an issue not encountered in the Toxoplasmosis Test Group or the Dual Test Group. It is important to note, however, that, notwithstanding this irregularity, the potential for a Type I Error remains minimal, since the effects of borreliosis on stress and anxiety did not reach statistical significance in the Borreliosis Test Group, as detailed below. Homogeneity of regression slopes was examined by testing interactions between covariates and grouping variables and was not violated in the reported models. Across the *Toxoplasma*, *Borrelia*, and combined *Toxoplasma*–*Borrelia* analyses, interactions involving the covariate (age) were generally non‐significant, indicating that the assumption of homogeneity of regression slopes was largely met. In the *Toxoplasma* and combined *Toxoplasma*–*Borrelia* models, a higher‐order *Toxoplasma* × sex × age interaction reached significance, suggesting a modest sex‐specific variation in the age–anxiety relationship within *Toxoplasma* groups. In contrast, all age‐related interactions in the *Borrelia*‐only analysis were non‐significant, fully supporting slope homogeneity. Overall, the homogeneity of regression slopes assumption can be considered sufficiently met, with only a minor, higher‐order deviation observed. Crucially, the nonparametric test (partial Kendall correlation with age and sex controlled performed separately for toxoplasmosis and borreliosis and men and women) provided practically identical results as parametric tests (MANCOVA, ANCOVA, and path analysis).

MANCOVA models were constructed for the dependent variables of anxiety and stress, and the independent variables of sex, either toxoplasmosis or borreliosis, or both toxoplasmosis and borreliosis, controlled for age.

The MANCOVA analysis for the Toxoplasmosis Test Group revealed significant main effects for toxoplasmosis and age, while the interaction effect between toxoplasmosis and sex was not significant. The MANCOVA modeling analysis for the Borreliosis Test Group revealed that neither borreliosis nor its interaction with sex was significant, whereas age and sex themselves had significant effects. MANCOVA results for the Dual Test Group demonstrated that the effect of toxoplasmosis was again significant, but the effect of borreliosis, the interactions between sex and toxoplasmosis, sex and borreliosis, toxoplasmosis and borreliosis, and toxoplasmosis, borreliosis, sex, and age proved non‐significant. For more details on all MANCOVA models see Table 2.

**TABLE 2:** Results of multivariate analysis of covariance for anxiety and stress.

Dual Test Group	Value	*F*_(2,504)_	*p*	Partial eta^2^
Intercept	0.608	391.206	**0.000**	0.608
Age	0.011	2.878	0.057	0.011
Sex	0.003	0.879	0.416	0.003
Toxoplasmosis	0.021	5.477	**0.004**	0.021
Borreliosis	0.008	2.038	0.131	0.008
Sex × Toxoplasmosis	0.007	1.650	0.193	0.007
Sex × Borreliosis	0.002	0.571	0.565	0.002
Toxoplasmosis × Borreliosis	0.002	0.400	0.670	0.002
Sex × Toxoplasmosis × Borreliosis	0.006	1.477	0.229	0.006

Toxoplasmosis Test Group	Value	*F*_(2,692)_	*p*	Partial eta^2^
Intercept	0.642	619.237	**0.000**	0.642
Age	0.015	5.143	**0.006**	0.015
Sex	0.003	1.106	0.331	0.003
Toxoplasmosis	0.014	5.051	**0.007**	0.014
Sex × Toxoplasmosis	0.006	2.260	0.105	0.006

Borreliosis Test Group	Value	*F*_(2,1578)_	*p*	Partial eta^2^
Intercept	0.690	1756.758	**0.000**	0.690
Age	0.014	11.432	**0.000**	0.014
Sex	0.028	22.924	**0.000**	0.028
Borreliosis	0.002	1.423	0.241	0.002
Borreliosis × Sex	0.001	0.713	0.490	0.001

*Note:* × indicates interaction; only the results for Pillai's Trace (“Value”) are reported in the table as Wilks' Lambda, Pillai's Trace, Hotelling's Trace, and Roy's Largest Root tests yielded the same results. The *p* shows the statistical significance of the MANCOVA tests; the statistically significant results *p* < 0.05 are printed in bold. For clarity, *p*‐values lower than 0.0005 are reported as 0.000 in the table. MANCOVA analyses showed a significant main effect of *Toxoplasma* infection on anxiety and stress in both the Toxoplasmosis and Dual Test Groups, while *Borrelia* infection had no significant effect in any group. Sex and age showed expected influences—women and younger participants tended to report higher anxiety and stress levels—but no significant interactions between infection and sex were detected.

The ANCOVA follow‐up univariate analysis for the Toxoplasmosis Test Group showed that the effects of toxoplasmosis and its interaction with sex were significant for anxiety, so that higher levels of anxiety were present in infected individuals, and, as the follow‐up analysis showed, infected men were the most affected. Although the toxoplasmosis effect also proved significant for stress showing that infected individuals experienced higher levels of stress, its interaction with sex did not. The ANCOVA follow‐ups for Borreliosis Test Group also revealed that neither borreliosis nor its interaction with sex were significant for anxiety, and the same held for stress. The ANCOVA results for the Dual Test Group indicated that, the effects of borreliosis, the interaction between toxoplasmosis and borreliosis, the interaction between borreliosis and sex, the interaction between toxoplasmosis and sex, and the interaction among borreliosis, toxoplasmosis, and sex were not significant for either anxiety or stress, these effect for toxoplasmosis were significant, specifically when anxiety was concerned. ANCOVA tests also showed that older participants expressed significantly lower anxiety and stress. For the strength, significance, and direction of the effects see Table 3.

**TABLE 3:** Results of univariate analysis of covariance for anxiety and stress.

	Anxiety				Stress			
	Dual Test Group				Dual Test Group			
	Sum of squares	*F*_(1,505)_	*p*	Partial eta^2^	Sum of squares	*F*_(1,505)_	*p*	Partial eta^2^
Intercept	77243.301	698.096	**0.000**	0.580	30199.939	746.167	**0.000**	0.596
Toxoplasmosis	1061.081	9.590	**0.002**	0.019	162.159	4.007	**0.046**	0.008
Borreliosis	86.304	0.780	0.378	0.002	1.865	0.046	0.830	0.000
Sex	36.366	0.329	0.567	0.001	50.838	1.256	0.263	0.002
Age	605.185	5.469	**0.020**	0.011	208.949	5.163	**0.023**	0.010
Toxoplasmosis × Borreliosis	43.237	0.391	0.532	0.001	1.461	0.036	0.849	0.000
Toxoplasmosis × Sex	342.224	3.093	0.079	0.006	121.940	3.013	0.083	0.006
Borreliosis × Sex	123.584	1.117	0.291	0.002	26.479	0.654	0.419	0.001
Toxoplasmosis × Borreliosis × Sex	105.449	0.953	0.329	0.002	0.239	0.006	0.939	0.000
Residuals	55877.545				20439.071			

	Anxiety				Stress			
	Toxoplasmosis Test Group				Toxoplasmosis Test Group			
	Sum of squares	*F*_(1,693)_	*p*	Partial eta^2^	Sum of squares	*F*_(1,693)_	*p*	Partial eta^2^
Intercept	122770.733	1125.627	**0.000**	0.619	48007.854	1152.585	**0.000**	0.625
Toxoplasmosis	1000.727	9.175	**0.003**	0.013	167.772	4.028	**0.045**	0.006
Sex	14.717	0.135	0.713	0.000	50.003	1.200	0.274	0.002
Age	1083.466	9.934	**0.002**	0.014	367.678	8.827	**0.003**	0.013
Toxoplasmosis × Sex	493.493	4.525	**0.034**	0.006	129.354	3.106	0.078	0.004
Residuals	75584.626				28865.067			

	Anxiety				Stress			
	Borreliosis Test Group				Borreliosis Test Group			
	Sum of squares	*F*_(1,1579)_	*p*	Partial eta^2^	Sum of squares	*F*_(1,1579)_	*p*	Partial eta^2^
Intercept	289756.175	2966.591	**0.000**	0.653	121187.212	3387.170	**0.000**	0.682
Borreliosis	133.262	1.364	0.243	0.001	97.555	2.727	0.099	0.002
Sex	3435.659	35.175	**0.000**	0.022	1626.718	45.467	**0.000**	0.028
Age	1126.663	11.535	**0.001**	0.007	791.621	22.126	**0.000**	0.014
Borreliosis × Sex	77.857	0.797	0.372	0.001	3.010	0.084	0.772	0.000
Residuals	154225.844				56493.943			

*Note:* × indicates interaction. The *p* shows the statistical significance of the ANCOVA tests; the statistically significant results *p* < 0.05 are printed in bold and *p*‐values lower than 0.0005 are reported as 0.000 in the table. ANCOVA showed that toxoplasmosis increased anxiety and stress, with a significant toxoplasmosis × sex interaction for anxiety (infected men most affected). In the Dual and Borreliosis Test Groups, neither main effects nor interactions were significant. Age correlated negatively with both outcomes, and sex affected stress only in the Borreliosis group.

The significant multivariate effects of toxoplasmosis and the weaker univariate effects for stress reflect the different hypotheses tested by MANCOVA and ANCOVA. MANCOVA evaluates group differences in the joint multivariate outcome space, taking into account the correlation between dependent variables, whereas univariate ANCOVAs assess each outcome separately.

Anxiety and stress are conceptually and empirically correlated constructs, and toxoplasmosis showed effects in the same direction on both variables. Even when the effect on stress was relatively small, its covariance with anxiety increased sensitivity at the multivariate level. Consequently, the MANCOVA detected a reliable overall shift in the anxiety–stress profile associated with toxoplasmosis, despite the fact that anxiety accounted for the majority of the effect. These patterns indicate that toxoplasmosis is more strongly related to anxiety than to stress per se, and the multivariate results should not be interpreted as evidence of equally strong effects on both outcomes.

#### 3.2.2 Could Impaired Physical Health Mediate the Effects of Infections on Stress and Anxiety? (Mediation Analyses Using Partial Kendall Correlations)

The Stress‐Coping Hypothesis suggests a mediating role of physical health and stress in the effects of toxoplasmosis on behavioral variables and personality traits. Concurrently, the data presented in Table 1 indicate that physical health is indeed influenced by toxoplasmosis. To assess this hypothesis and at the same time to confirm the results of parametric tests with more robust (assumption‐free) nonparametric tests, we analyzed the effects of infections on stress and anxiety using partial Kendall correlation test. To explore whether physical health impairment might account for the observed associations, we juxtaposed the outcomes of analyses that controlled solely for age and sex against those that also controlled for physical health, observing any attenuation consistent with potential mediation (Table 4).

**TABLE 4:** Partial Kendall Tau correlations and their attributed *p*‐values.

Dual Test Group		Covariates age and sex						Covariates physical health, age and sex					
		All		Women		Men		All		Women		Men	
		Anxiety	Stress	Anxiety	Stress	Anxiety	Stress	Anxiety	Stress	Anxiety	Stress	Anxiety	Stress
Toxoplasmosis	*τ*	0.086	0.054	0.053	0.025	0.171	0.132	0.058	0.029	0.032	0.007	0.122	0.088
	*p*	**0.003**	0.063	0.129	0.464	**0.002**	**0.016**	**0.048**	0.314	0.349	0.833	**0.027**	0.111
Borreliosis	*τ*	0.006	0.044	0.006	0.05	0.008	0.022	−0.015	0.027	−0.021	0.029	0.003	0.018
	*p*	0.814	0.134	0.845	0.152	0.879	0.681	0.602	0.356	0.547	0.407	0.956	0.736

Toxoplasmosis Test Group		Covariates age and sex						Covariates physical health, age and sex					
		All		Women		Men		All		Women		Men	
		Anxiety	Stress	Anxiety	Stress	Anxiety	Stress	Anxiety	Stress	Anxiety	Stress	Anxiety	Stress
Toxoplasmosis	*τ*	0.068	0.042	0.044	0.02	0.141	0.114	0.044	0.021	0.024	0.002	0.106	0.084
	*p*	**0.007**	0.095	0.123	0.478	**0.006**	**0.026**	0.079	0.404	0.407	0.941	**0.039**	0.102

Borreliosis Test Group		Covariates age and sex						Covariates physical health, age and sex					
		All		Women		Men		All		Women		Men	
		Anxiety	Stress	Anxiety	Stress	Anxiety	Stress	Anxiety	Stress	Anxiety	Stress	Anxiety	Stress
Borreliosis	*τ*	0.026	0.034	0.013	0.023	0.051	0.047	0.02	0.03	0.011	0.021	0.042	0.04
	*p*	0.118	**0.041**	0.538	0.285	0.051	0.068	0.211	0.073	0.605	0.316	0.109	0.121

*Note:* Positive Kendall Tau partial correlation coefficients indicate a rise in anxiety or stress levels in association with toxoplasmosis or borreliosis infection. Conversely, negative coefficients signify a reduction in anxiety or stress levels correlated with these infections. Significant *p* values are highlighted in bold. Partial Kendall correlations confirmed that toxoplasmosis is linked to higher anxiety and stress, especially in men, but these effects disappeared after controlling for physical health, indicating that the associations were significantly attenuated when physical health was included in the model, a pattern consistent with the hypothesized mediation via health deterioration. Borreliosis showed no significant associations with stress or anxiety after controlling for this variable.

The initial, less complex model without control for physical health for the Toxoplasmosis Test Group demonstrated that toxoplasmosis significantly impacts anxiety. The associations between toxoplasmosis and both stress and anxiety were stronger and statistically significant in men, while in women, they were weaker and did not reach significance. When the model was expanded to include the covariates of age, sex, and physical health, the observed effects of toxoplasmosis diminished. Only the impact of the infection on anxiety in men remained significant. Similar analyses for borreliosis did not identify any significant effect except for its correlation with stress across the entire sample, including both men and women. Again, the significant correlation and also trends observed were significantly attenuated when physical health was controlled (see Table 4).

#### 3.2.3 Direct and Indirect Effects of Infections on Health, Stress, and Anxiety (Path Analysis)

**FIGURE 2:**
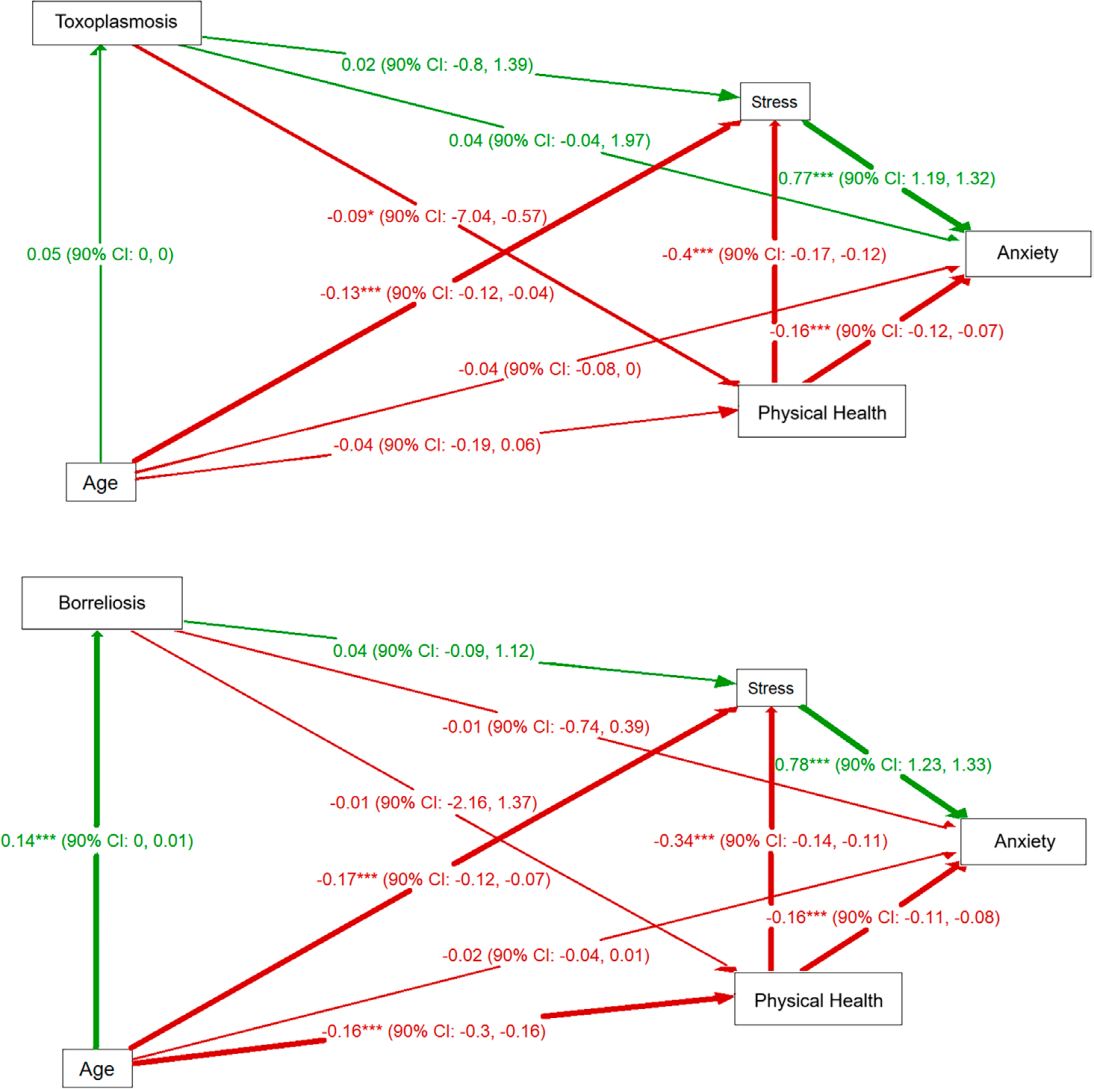
Path analysis for Toxoplasmosis Test and Borreliosis Test groups. Positive path coefficients (green arrows) indicate that an increase in the source variable leads to an increase in the dependent variable. Conversely, negative coefficients (red arrows) imply that an increase in the source variable results in a decrease in the dependent variable. For dichotomous variables like toxoplasmosis and borreliosis, positive coefficients indicate that infection is associated with an increase in the dependent variable, while negative coefficients indicate that infection is associated with a decrease in the dependent variable. *p < 0.05; **p < 0.01; ***p < 0.001. Path analysis revealed that toxoplasmosis affected stress and anxiety only indirectly, mainly through impaired physical health and stress mediation, whereas borreliosis showed no significant direct or indirect effects on either variable.

**FIGURE 3:**
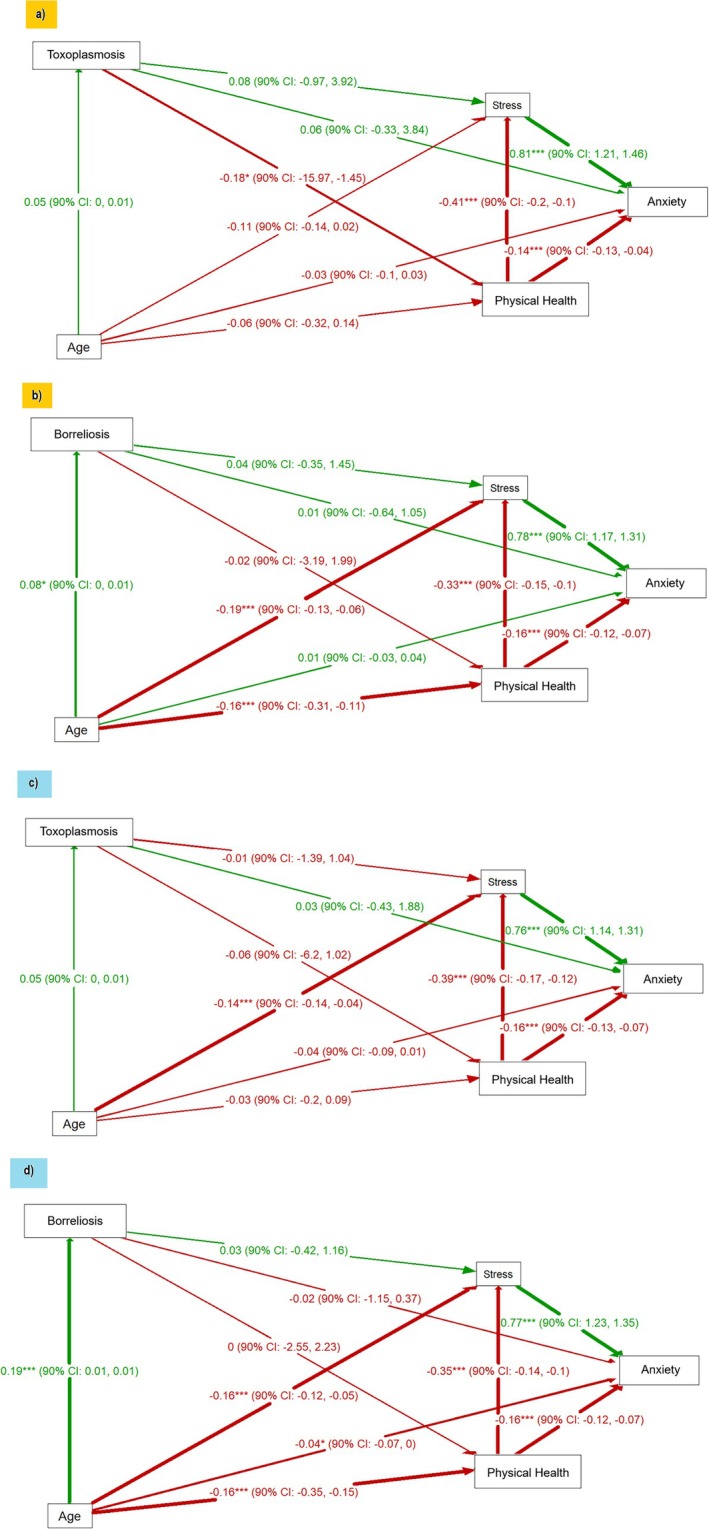
Path analysis for men and women. Two sets of path diagrams are presented: Models for men are shown in parts a and b (labeled in orange), and models for women are in parts c and d (labeled in blue). Please consult the legend for Figure 2 for a detailed explanation. When analyzed separately by sex, toxoplasmosis shows a stronger indirect effect on stress and anxiety in men than in women, mediated mainly via worse physical health. Borreliosis shows no significant effects on stress or anxiety in either sex. In the borreliosis models, age has a strong negative effect on physical health, consistent across men and women.

The outcomes of the partial Kendall analysis indicate that physical health plays a pivotal role in mediating the correlations of infections with stress and anxiety. However, this analysis neither elucidates the mechanisms behind such mediation nor excludes the possibility that the infections may also have direct effects on stress or its significant psychological symptom, anxiety. To further investigate these critical issues, we employed structural equation modeling, specifically path analysis. The impacts of toxoplasmosis and borreliosis were analyzed separately. The models included variables such as age, physical health, anxiety, stress, and infection status (toxoplasmosis or borreliosis). In additional post hoc analyses, the effect of sex was evaluated by conducting identical analyses for each sex separately. The findings depicted in Figures 2 and 3 suggest that toxoplasmosis exerts only indirect effects on stress, these being mediated through its direct impacts on physical health. The effect of toxoplasmosis on anxiety also seems to be mostly indirect, mediated through physical health. However, a substantial portion of the effect of toxoplasmosis on anxiety can be ascribed to its physical health‐mediated influence on stress and the pronounced impact of stress on anxiety. Post hoc analyses indicate that this pattern slightly differs between men (Figure 3a) and women (Figure 3c). In women, the physical health‐mediated effect of toxoplasmosis on stress is nonsignificant and much weaker than in men, and its direct effect on stress is even weaker (and negative instead of positive).

In contrast, borreliosis exerts a significant direct effect on stress, and a weak nonsignificant effect on physical health. Here, the overarching trend observed is strikingly similar in men (Figure 3b) and women (Figure 3d). For a more detailed interpretation of the results of path analyses see Discussion.

## 4 Discussion

The combined use of partial Kendall correlations and path analysis allowed us to disentangle direct and indirect effects of *Toxoplasma* and *Borrelia* infections on stress and anxiety while controlling for sex, age, and physical health. Across both approaches, the results consistently supported the Stress–Coping Hypothesis: the association between toxoplasmosis and stress was primarily mediated by impaired physical health rather than by a direct psychological effect. Physical health showed a robust negative relationship with stress, which in turn strongly predicted anxiety. The direct path from *Toxoplasma* infection to stress was weak and nonsignificant once physical health was included in the model. For *Borrelia*, no significant effects on either stress or anxiety were detected. Overall, the alignment between the non‐parametric and structural models increases confidence in the robustness of these findings. A more detailed interpretation of model components and sex‐specific patterns follows in the subsequent sections.

The observed link between impaired physical health, stress, and anxiety is consistent with current neuroimmunological and psychosocial models describing reciprocal feedbacks between physiological and psychological processes. The findings fit a mechanistic cascade in which latent infection contributes to chronic health impairment that elevates perceived stress; stress, in turn, feeds back on immune and neuroendocrine function (e.g., HPA‐axis tone and glucocorticoid sensitivity), shaping neuromodulatory systems involved in affect regulation. These processes are plausibly sex‐modulated (beyond testosterone alone) through differences in immune reactivity, hormone signaling, and receptor profiles. Crucially, the relationship can form a positive feedback loop: infection‐related health problems increase stress; stress can suppress or dysregulate immune function; impaired immunity heightens susceptibility to additional or more severe health issues. This, in turn, reinforces stress and a range of psychological and neuropsychiatric symptoms repeatedly documented in individuals with latent toxoplasmosis (Latifi and Flegr 2025). Within this framework, the Stress–Coping Hypothesis is consistent with an indirect pathway from infection to anxiety primarily via health and stress, with sex psychobehavioral differences largely emerging from the modulation of these interacting systems.

### 4.1 Association of *Toxoplasma* Infection With Higher Stress and Anxiety

Using standard psychological questionnaires; namely, the STAI‐X2 and PSS, we measured perceived stress and anxiety in 1768 men and women who had previously undergone laboratory testing for toxoplasmosis or borreliosis and provided information about the results of these tests. The collected data were used in our study to test the Stress‐Coping Hypothesis (Lindová et al. 2010, 2006). This hypothesis suggests that many psychological differences between individuals infected with the *Toxoplasma* parasite, particularly those that are directionally opposite in men and women, are not likely the result of the parasite's manipulative activity aimed at facilitating transmission from the infected to the uninfected host but are induced by chronic stress caused by the long‐term impact of the infection on physical health. The outputs of both parametric and non‐parametric tests were consistent with the Stress‐Coping Hypothesis (Lindová et al. 2010, 2006). While the direct effect of infection on stress and, consequently, on its primary psychological manifestation, anxiety, was relatively weak, toxoplasmosis had a strong effect on physical health. Deterioration in physical health had a direct effect on stress, resulting in higher levels of stress (and its manifestation, anxiety) in *Toxoplasma*‐infected individuals. In our sample, both direct and indirect effects of toxoplasmosis were stronger in men than in women.

### 4.2 Association of *Borrelia* Infection With Higher Stress and Anxiety

Although the public is more concerned about the long‐term health consequences of borreliosis than toxoplasmosis, the association of borreliosis with physical health was minimal (non‐existent in women) and the observed weaker positive association between borreliosis and anxiety in men was likely due to a higher incidence of toxoplasmosis in individuals with borreliosis, as discussed below. This confirms that the impact of toxoplasmosis is likely specific and, from a public health perspective, more significant than the influence of other similarly widespread chronic infections. This conclusion is in line with the results of previously published studies. These have shown that differences in the prevalence of toxoplasmosis explain 23% of the variance in overall disease burden in different European countries (Flegr et al. 2014) and that the prevalence of toxoplasmosis is the third most important predictor of the ratio of male to female births (with a lower ratio serving as a proxy for impaired health) among the 15 factors studied (Dama et al. 2016).

The absence of associations between infection and health in the case of borreliosis calls into question the notion that the connection between infection and health is simply an artifact of questionnaire methodologies. It specifically casts doubt on the notion that individuals with heightened health awareness are predisposed to report worse health and the presence of an infection, thereby supporting the likelihood that, on average, *Toxoplasma*‐infected individuals truly experience poorer health and, secondarily, higher stress compared to those uninfected.

### 4.3 Why the Association of Toxoplasmosis With Anxiety Seems Stronger Than With Stress?

The overall (direct + indirect) association of toxoplasmosis with stress was consistently weaker than with anxiety. This seemingly contradicts our hypothesis, which posits that the association with stress is primary and that with anxiety is secondary, mediated by the impact of stress on anxiety (Hussenoeder et al. 2022). In reality, the strength of a statistical association—reflected in measures such as partial eta^2^ values, absolute Kendall Tau values, or absolute path coefficients—is influenced not only by the true strength of the association but also by the precision with which the studied variables are measured. In our study, the State–Trait Anxiety Inventory‐Revised X2, which consists of 20 questions, demonstrated higher reliability (Omega = 0.94) compared to the Perceived Stress Scale that contains only 10 questions (Omega = 0.90). This finding suggests that the STAI‐X2 may more accurately estimate psychological states than the PSS. Consequently, the measured strength of the association of infection with stress could appear lower than with anxiety in our analysis, even if the actual strength of the association with stress is higher.

### 4.4 Why Effects of Toxoplasmosis Were Stronger in Men Than in Women?

The strength of the association between toxoplasmosis and other variables was stronger in men than in women. This aligns with the results of most previous studies (Flegr and Horáček 2020; Flegr and Hrdý 1994; Flegr et al. 2018; Lindová et al. 2012; Příplatová et al. 2014). In the case of the association of latent toxoplasmosis with morphological and some behavioral traits, this could be due to the physiological mechanism of some behavioral effects of toxoplasmosis.

Experiments with laboratory‐infected rats have demonstrated that *Toxoplasma* infection often increases testosterone synthesis in the testes, leading to elevated testosterone levels which are directly or indirectly responsible for the observed behavioral changes. In female rats and castrated male rats, such an increase in testosterone levels and the corresponding behavioral changes do not occur (Lim et al. 2013). Increased testosterone levels have also been observed in *Toxoplasma*‐infected men, whereas infected women exhibited lower testosterone levels (Flegr et al. 2008). The higher stress and greater intensity of stress‐dependent psychological changes in men could be related to an increased testosterone level. High levels of testosterone could lead to immunosuppression and therefore more frequent illnesses, which could deteriorate physical health, increase stress levels, and secondarily increase anxiety. The health deterioration observed, although to a lesser extent, in infected women suggests that *Toxoplasma* impacts physical health and thereby stress also through mechanisms beyond just elevated testosterone synthesis in the testes.

Of course, it is possible that the greater intensity of *Toxoplasma*‐associated manifestations in men compared to women could have entirely different causes. Most women learned of their *Toxoplasma* positivity during preventive screening in pregnancy, whereas men were tested for toxoplasmosis while investigating possible causes of health issues. For example, among 2044 participants of a similar internet study, 37.6% women were screened for toxoplasmosis in relation to their pregnancy, 40.3% men and 34.3% women were tested in our lab during participation in various research projects, and 49.4% men and 20.7% women were tested in relation to their health problems (Flegr and Preiss 2019). Therefore, given that men were more than twice as likely as women to be tested for toxoplasmosis in relation to health problems, it is probable that, on average, male participants suffered from a clinically more severe form of toxoplasmosis than female participants.

It can be also hypothesized that due to different stress‐coping strategies in men and women, the questionnaires may measure stress and anxiety more accurately in men than in women. When testing the reliability of each questionnaire separately for men and women, we found the results to be nearly identical across genders, although the reliability differed between the two questionnaires, see the Materials and Methods section. However, it is important to acknowledge that a questionnaire's statistical reliability does not automatically guarantee its construct validity. This means a questionnaire intended to measure anxiety or stress might not do so with equal validity for both men and women. This discrepancy arises because men and women typically encounter different stressors, utilize distinct coping strategies, are socialized into different narratives around anxiety and stress (McLean and Anderson 2009), and may interpret the same questionnaire item differently.

### 4.5 Do Some Symptoms of Senescence Actually Reflect the Increasing Prevalence of Toxoplasmosis in Age Strata?

Path analysis revealed that age, unsurprisingly, correlates positively with the likelihood of having been diagnosed with borreliosis and toxoplasmosis during one's lifetime. However, a potentially significant finding emerged from comparing the correlation of age with physical health in the path analysis model including toxoplasmosis and the model including borreliosis. In the model concerning borreliosis—a condition which had no effect on physical health—a strong negative impact of age on physical health was observed. In the model incorporating toxoplasmosis instead of borreliosis—where toxoplasmosis effects were explicitly controlled—the impact of age on physical health was observed to be four times weaker. This suggests that the influence of age on physical health, commonly seen as aging manifestations, might be significantly impacted by toxoplasmosis. Given the cumulative probability of having contracted toxoplasmosis over time, and its latent manifestations often being cumulative (Flegr et al. 2005, 1996; Flegr and Hrdý 1994; Lindová et al. 2012), it is conceivable that some health deteriorations commonly attributed to aging could actually result from the pathological effects of toxoplasmosis.

The public health impact of toxoplasmosis on the whole population could be substantial. On average, about one‐third of the population is infected with *Toxoplasma*. Given that its prevalence increases with age, it is likely that more than half of the population in older age groups is infected. Moreover, in older infected humans, the level of anamnestic anti‐*Toxoplasma* IgG antibodies decreases in some individuals below the positivity threshold. This is manifested, for example, by the fact that although the seroprevalence of toxoplasmosis should increase with age, it only rises up to a certain age and then begins to decrease in the respective age groups (Flegr 2017; Kolbeková et al. 2007). This indicates that the prevalence values commonly reported, particularly for the oldest age groups, are much lower than the actual prevalence of infected individuals within those age categories.

### 4.6 Are Our Findings Regarding Significantly Worse Health of Individuals With Latent Toxoplasmosis Consistent With Current Knowledge?

Independent partial clinical studies and extensive systematic research, primarily conducted in the last decade, indeed show that the prevalence of latent toxoplasmosis significantly impacts public health; for a review, see (Flegr et al. 2014). For example, a comprehensive systematic cross‐sectional online study, which examined the incidence of all disease types, reveals that a significant portion of diseases occur more frequently in individuals diagnosed with latent toxoplasmosis compared to those without the infection. Specifically, the study showed that, of the 134 disorders reported by at least 10 participants, 77 were significantly more common in *Toxoplasma*‐infected participants than in *Toxoplasma*‐free participants. The study also revealed that 333 infected subjects scored significantly worse than 1153 uninfected controls in 28 of 29 health‐related variables (Flegr and Escudero 2016). Similar results were provided by a large ecological study that examined the correlation of the prevalence of toxoplasmosis in 88 countries with health impacts, specifically age‐standardized Disability Adjusted Life Year (DALY), for 128 diseases monitored by WHO over the long term (Flegr et al. 2014). The study showed that 23 of these diseases statistically significantly correlate with the prevalence of toxoplasmosis in individual countries. Such diseases include major public health concerns such as inflammatory heart disorders, ischemic heart disease, cerebrovascular disease, prostate cancer, and epilepsy. The study also showed that differences in the occurrence of toxoplasmosis in individual countries, after filtering out confounding variables such as Gross Domestic Product per capita (GDP), geolatitude, and humidity, explain 23% of all variability in overall morbidity in the 29 European countries included in the study.

### 4.7 Possible Reasons for a Positive Association Between *Toxoplasma* and *Borrelia* Infection

An incidental finding of the current study was the demonstration of a strong correlation between *Toxoplasma* and *Borrelia* infections; individuals previously diagnosed with one parasite had a significantly higher probability of being diagnosed with the other. This positive association may explain the seemingly direct positive association of borreliosis with stress (path coefficient = 0.04, *p* = 0.032). Most likely, a positive test for borreliosis simply indicated a higher likelihood of toxoplasmosis positivity. Toxoplasmosis was not included in the model, and moreover, the majority of study participants were not tested for toxoplasmosis. Although the analysis might suggest that borreliosis positivity is associated with worsened health, it was actually toxoplasmosis, not borreliosis, that impacted physical health and subsequently increased stress.

A positive association between *Toxoplasma* and *Borrelia* seropositivity was also reported in previous studies (Flegr and Horáček 2018; Flegr et al. 2023). The existence of this association can be explained in at least three fundamentally different ways. The first possibility is that infection by one of the pathogens could increase the likelihood of infection by the other pathogen, most likely through the induction of changes in the host's immune system functioning—immunomodulation leading to reduced responsiveness or non‐responsiveness to certain types of antigens. Indeed, numerous studies have shown that toxoplasmosis induces dramatic changes in the immune system functioning of both infected animals and humans (Flegr and Stříž 2011; Kaňková et al. 2010; Tomita et al. 2021).

The second possibility is that both pathogens could be transmitted to humans simultaneously—for instance, through tick bites. Borreliosis in the Czech Republic is transmitted by *Ixodes ricinus* ticks. While this mode of transmission is not yet assumed for toxoplasmosis, *Toxoplasma* DNA has been identified in *Ixodes ricinus* at a rate almost identical to *Borrelia* (12.6% vs. 12.7%) (Sroka et al. 2009). Moreover, both pathogens were detected together in 2.3% of all ticks and 3.8% of adult female ticks. Previous research has also confirmed the transmission of *Toxoplasma* by three tick species: *Dermacentor variabilis* , *D. andersoni* , and *Amblyomma americanum* (Woke et al. 1953).

The third possibility is that the association could be an artifact of questionnaire studies—a portion of individuals might be more likely to respond positively to both questions, even though they are not actually infected with both pathogens. We attempted to mitigate this risk in the questionnaire. The participants were explicitly asked to report only laboratory test results regarding their infection status with the specified pathogens, and the questionnaire was preset to indicate as a default the response “I do not know, I am not sure, I have not been tested.” Even so, it is possible that individuals with certain psychological dispositions, such as heightened concerns about their health, perhaps akin to hypochondria, may have tended to answer both questions positively. Therefore, our potentially significant findings should be considered only preliminary until the existence of an association between borreliosis and toxoplasmosis is confirmed through a study in which both infections are diagnosed serologically during the study itself.

### 4.8 Strengths and Limitations of the Study

The main strength of this study is the large number of participants and the fact that they were not pre‐informed that the questionnaire would also contain questions on toxoplasmosis and borreliosis. The entire study, conducted during the first year of the COVID‐19 pandemic, was primarily focused on the impact of COVID‐19 and pandemic measures on physical health and wellbeing, with the two questions regarding infections by *Toxoplasma* and *Borrelia* placed towards the end of the questionnaire. Thus, participants could not consciously or subconsciously bias the results based on their beliefs about the influence of toxoplasmosis on their physical health.

A significant limitation of the study is the self‐selection of participants, which means our sample does not represent a random cross‐section of the general population. Comparison with the 2021 Czech National Census data reveals a notable demographic skew in our convenience sample. While the national population is approximately 51% female with a median age of 42.7 years, our participants were predominantly female (59%–75% depending on the sample) and notably younger, with mean ages between 31 and 36 years. Furthermore, our recruitment via social media likely favored individuals with higher engagement in health topics or greater survey willingness, resulting in a higher proportion of university‐educated individuals than the 19% national average reported by the Czech Statistical Office. These features limit the generalizability of the findings to the broader Czech population. However, it is important to emphasize that the self‐selection of participants was likely random with respect to *Toxoplasma* and *Borrelia* infection, as participants were not aware that the questionnaire would include questions on these variables. Due to these demographic discrepancies, we recommend exercising caution when generalizing our conclusions to the broader Czech population, as the internal mechanisms observed in this cohort may be influenced by its specific socioeconomic and age profile. Another limitation is that the anonymous study participants self‐reported their laboratory test results for toxoplasmosis and borreliosis, and this information could not be verified. At least in the case of borreliosis, such self‐reported information is more accurate than if participants had been tested in the study, as levels of anti‐*Borrelia* antibodies often disappear or fluctuate significantly over time after infection. To what extent the same applies to toxoplasmosis is not clear, but reversion to seronegative status is occasionally reported in the literature. However, our results, specifically the decreasing seroprevalence of toxoplasmosis in middle and old age groups, suggest that such reversions might be relatively frequent (Flegr 2017). It is practically certain that some study participants provided incorrect or outdated data about their infection status; however, the representation of these individuals in the samples is likely very low, according to the findings of one of our earlier studies. In this online study, 1865 respondents had the option to provide their code, under which they had participated in our studies in the past. This option was utilized by 393 individuals. Verification showed that in 99.2% of cases, the *Toxoplasma* status reported in the questionnaire matched the *Toxoplasma* status determined by our serological tests (Flegr 2017). Another study demonstrated that the effects of toxoplasmosis on physical and mental health detected in the entire sample of 6397 individuals were practically identical in strength and direction to those detected in a subsample of 800 individuals who provided their names at the end of the questionnaire, allowing their *Toxoplasma* status to be verified (Flegr and Horáček 2020).

An additional limitation is that the study was conducted during a pandemic, a time likely marked by increased levels of stress and anxiety within the population. At the beginning of the study and several times throughout the questionnaire, participants were specifically asked to assess their physical health during the ongoing pandemic. For this reason, it is not possible to compare the results obtained during this study with those from normal circumstances, making the extent to which the findings are generalizable uncertain. Furthermore, it is conceivable that our findings do not indicate increased stress and anxiety in *Toxoplasma*‐infected subjects but rather imply a diminished capacity in *Toxoplasma*‐positive individuals to cope with stressful situations, like the COVID‐19 pandemic. It is even possible that the infected individuals objectively face a more severe progression of COVID‐19 infection compared to individuals without *Toxoplasma* infection (Flegr 2021), and that the observed increase in stress and health problems among infected participants could be a consequence of more severe COVID‐19 symptoms rather than a direct effect of *Toxoplasma* infection itself.

Another factor further limiting the generalizability of our findings is the likely characteristics of our participants with regard to the instruments used for data collection. The study relied on volunteers who completed a relatively long online questionnaire without any financial or material compensation. This self‐selecting group likely consisted of individuals with higher interest in science, health, or psychological topics and therefore may not fully represent the general population. Together with the specific circumstances of data collection during the COVID‐19 pandemic and the use of online recruitment, this limits the extent to which our results can be generalized beyond this study to a certain degree.

Our objective was to assess the impact of infections on long‐term psychological states rather than immediate feelings. Hence, we utilized the STAI‐X2 variant, which measures anxiety as a trait. However, our questionnaire also included the STAI‐6, a brief 6‐item inventory that assesses immediate feelings (state) (Marteau and Bekker 1992). To verify the robustness of our results, we performed analyses with the STAI‐6 as an alternative to the STAI‐X2; the outcomes were nearly identical (refer to Figure S1). This consistency implies that, within the current study's context, whether anxiety is measured as a trait or a state appears not to be critically important.

In addition to reporting the results of testing our main hypothesis (higher stress levels in *Toxoplasma*‐infected individuals), we also present findings from supplementary exploratory analyses. These additional analyses were not preregistered and may therefore be affected by multiple testing artifacts. Consequently, their results should be considered preliminary and require confirmation in independent datasets.

## 5 Conclusion

The Stress‐Coping Hypothesis published nearly 20 years ago has so far only been tested and confirmed for one of its two predictions: the deteriorated physical health of individuals with latent toxoplasmosis. Despite an extensive literature review, no published study was found testing its second prediction, increased stress levels in infected individuals. Our study addresses this gap, showing that infected individuals exhibit significantly higher stress and anxiety levels than uninfected ones. The study also reveals that the impact of toxoplasmosis on anxiety and stress is largely mediated by deteriorating physical health in infected individuals. Comparisons of results for toxoplasmosis and borreliosis further indicate that the association between infection, health, and consequently stress is specific to toxoplasmosis and not observed in borreliosis. Furthermore, these comparisons suggest that numerous changes in physical condition commonly attributed to aging could, in fact, result from the increasing prevalence of toxoplasmosis with age and the cumulative effects of latent toxoplasmosis.

Our current study strengthens the conclusions of recent research, emphasizing the significant public health impact of latent toxoplasmosis in comparison to other latent infections. Today, around a third of humanity is infected with *Toxoplasma*, and the majority in both developing and developed countries will be infected over their lifetimes. There is no effective drug available for the treatment of lifelong latent toxoplasmosis in human medicine. However, since *Toxoplasma* can only sexually reproduce in felines, it could be entirely eradicated from the human population using oral vaccines administered to domestic and feral cats. The findings of studies, including ours, suggest that developing such vaccines should be a priority.

## Author Contributions

J.F. and Š.K. conceived and designed the study. J.F. collected the data and prepared the initial draft of the manuscript. A.L. performed the data analyses. All authors contributed to manuscript revision and approved the final manuscript.

## Funding

This research was supported by the Czech Science Foundation, grant number 22‐20785S.

## Disclosure

The article contains no material from other sources.

## Ethics Statement

The study was approved by the Institutional Review Board of the Faculty of Science, Charles University (no. 2020/25), and conducted in accordance with all relevant guidelines and regulations. All participants provided informed consent.

## Conflicts of Interest

The authors declare no conflicts of interest.

## Supporting information


**Data S1:** sjop70085‐sup‐0001‐DataS1.docx.

## Data Availability

All data are available at figshare https://doi.org/10.6084/m9.figshare.25188788.v1.
