# Supplementary material for: From Infection to Anxiety: A Sequential Model Linking Latent Toxoplasmosis to Psychological Distress via Health and Stress
Source: Scand J Psychol. 2026 Feb 26;67(4):1026–43. doi: 10.1111/sjop.70085 (PMC13352548; doi:10.1111/sjop.70085)
Supplement: Supplementary file 1 — Data S1: sjop70085‐sup‐0001‐DataS1.docx. [file SJOP-67-1026-s001.docx]

Supplementary material:

Supplementary Table S1: Number of participants included in each statistical analysis by group

| Statistical Model | Group / Infection Status | Total N | Men N | Women N |
| --- | --- | --- | --- | --- |
| Descriptive Statistics | Toxoplasmosis Test Group | 698 | 172 | 526 |
|  | Borreliosis Test Group | 1,584 | 650 | 934 |
|  | Dual Test Group | 514 | 148 | 366 |
| MANCOVA/ ANCOVA | Toxoplasmosis Test Group | 698 | 172 | 526 |
|  | Borreliosis Test Group | 1,584 | 650 | 934 |
|  | Dual Test Group | 514 | 148 | 366 |
| Kendall Correlations | Toxoplasmosis Test Group | 698 | 172 | 526 |
|  | Borreliosis Test Group | 1,584 | 650 | 934 |
|  | Dual Test Group | 514 | 148 | 366 |
| Path Analysis | Toxoplasmosis Model | 698 | 172 | 526 |
|  | Borreliosis Model | 1,584 | 650 | 934 |

*The table shows the exact number of participants in each model. Small variations in N (e.g., 1,581 vs. 1,584 for Borreliosis) are due to occasional missing data in specific variables.*

Supplementary Table S2: Post hoc exploration of MANCOVA and ANCOVA results using univariate nonparametric tests

|  | Age | Physical health | Anxiety | Stress |
| --- | --- | --- | --- | --- |
| **Dual Test Group** | | | | |
| Toxoplasmosis (yes/no) | | | | |
| W | 19886 | 15068 | 21418 | 20338 |
| p | 0.238 | **0.007** | **0.016** | 0.123 |
| Borreliosis (yes/no) | | | | |
| W | 27157 | 18798 | 21867 | 23239 |
| p | **0.0001** | **0.037** | 0.859 | 0.235 |
| Sex(male/female) | | | | |
| W | 29160 | 27173 | 22463 | 22284 |
| p | 0.157 | 0.914 | **0.002** | **0.001** |
| **Toxoplasmosis Test Group** | | | | |
| Toxoplasmosis (yes/no) | | | | |
| W | 43621 | 34941 | 45110 | 43351 |
| p | 0.124 | **0.013** | **0.026** | 0.158 |
| Sex (male/female) | | | | |
| W | 47816 | 44283 | 39041 | 38133 |
| p | 0.261 | 0.676 | **0.006** | **0.001** |
| **Borreliosis Test Group** | | | | |
| Borreliosis (yes/no) | | | | |
| W | 327148 | 264635 | 288242 | 289862 |
| p | **0.0001** | 0.103 | 0.256 | 0.185 |
| Sex (male/female) | | | | |
| W | 308375 | 303184 | 244978 | 238605 |
| p | 0.589 | 0.967 | **0.0001** | **0.0001** |

*The table presents the Wilcoxon rank-sum test statistics (w) and significance levels (p). Results significant at p < 0.05 are highlighted in bold. For more accurate results, refer to Table 4, which displays the outcomes of multivariate nonparametric tests (partial Kendall correlation test controlled for sex and age, or sex, age, and physical health).*

Supplementary Figure S1 Path analysis for Toxoplasmosis Test and Borreliosis Test Groups with STAI-X2 outputs substituted by STAI-6 outputs


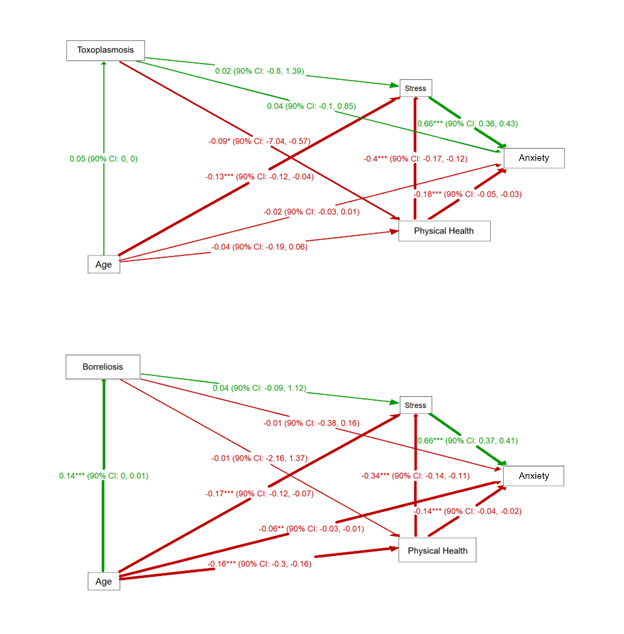


*Positive path coefficients (green arrows) indicate that an increase in the source variable leads to an increase in the dependent variable. Conversely, negative coefficients (red arrows) imply that an increase in the source variable results in a decrease in the dependent variable. For dichotomous variables like toxoplasmosis and borreliosis, positive coefficients indicate that infection is associated with an increase in the dependent variable, while negative coefficients indicate that infection is associated with a decrease in the dependent variable.*
** indicates p<0.05; ** indicates p<0.01; *** indicates p<0.001*
